# Improving the Prognosis of Colon Cancer through Knowledge-Based Clinical-Molecular Integrated Analysis

**DOI:** 10.1155/2021/9987819

**Published:** 2021-04-07

**Authors:** Danyang Tong, Yu Tian, Qiancheng Ye, Jun Li, Kefeng Ding, Jingsong Li

**Affiliations:** ^1^Engineering Research Center of EMR and Intelligent Expert System, Ministry of Education, College of Biomedical Engineering and Instrument Science, Zhejiang University, No. 38 Zheda Road, Hangzhou, 310027 Zhejiang Province, China; ^2^Department of Surgical Oncology, Second Affiliated Hospital, Zhejiang University School of Medicine, No. 88 Jiefang Road, Hangzhou, 31009 Zhejiang Province, China; ^3^Research Center for Healthcare Data Science, Zhejiang Lab, Hangzhou, China

## Abstract

**Background:**

Colon cancer has high morbidity and mortality rates among cancers. Existing clinical staging systems cannot accurately assess the prognostic risk of colon cancer patients. This study was aimed at improving the prognostic performance of the colon cancer clinical staging system through knowledge-based clinical-molecular integrated analysis.

**Methods:**

374 samples from The Cancer Genome Atlas Colon Adenocarcinoma (TCGA-COAD) dataset were used as the discovery set. 98 samples from the Clinical Proteomic Tumor Analysis Consortium (CPTAC) dataset were used as the validation set. After converting gene expression data into pathway dysregulation scores (PDSs), the random survival forest and Cox model were used to identify the best prognostic supplementary factors. The corresponding clinical-molecular integrated prognostic model was built, and the improvement of prognostic performance was assessed by comparing with the clinical prognostic model.

**Results:**

The PDS of 14 pathways played important roles in prognostic prediction together with clinical prognostic factors through the random survival forest. Further screening with the Cox model revealed that the PDS of the pathway hsa00532 was the best clinical prognostic supplementary factor. The integrated prognostic model constructed with clinical factors and the identified molecular factor was superior to the clinical prognostic model in discriminative performance. Kaplan-Meier (KM) curves of patients grouped by PDS suggested that patients with a higher PDS had a poorer prognosis, and stage II patients could be distinctly distinguished.

**Conclusions:**

Based on the knowledge-based clinical-molecular integrated analysis, a clinical-molecular integrated prognostic model and corresponding nomogram for colon cancer overall survival prognosis was built, which showed better prognostic performance than the clinical prognostic model. The PDS of the pathway hsa00532 is a considerable clinical prognostic supplementary factor for colon cancer and may represent a potential prognostic marker for stage II colon cancer. The PDS calculation involves only 16 genes, which supports its potential for clinical application.

## 1. Introduction

Colon cancer is one of the top cancers in terms of incidence and mortality in both China and America [1, 2]. Recent global surveillance of cancer trends revealed that further research on colon cancer is needed, as the age-standardized 5-year net survival of colon cancer ranges from approximately 15% to 75% in different countries [3].

Currently, the tumor, node, and metastasis (TNM) stage system proposed by the American Joint Committee on Cancer (AJCC) is the most commonly used clinical staging tool for colon cancer. However, the accuracy of the 7th TNM staging system for assessing the prognostic risk of colorectal cancer patients still needs to be improved, especially for stage II and stage IIIA patients [4]. The 8th edition of the TNM staging system was aimed at building an important bridge from a “population-based” to a more “personalized” approach to cancer stage [5]. The 8th edition of the TNM staging system for breast cancer did so by including the HER2 and ER statuses in its prognostic staging [6]. Several studies claimed that modifications of the TNM staging system for colorectal cancer showed improved prognostic performance [4, 7, 8]. However, no structural changes were made in the 8th edition of the TNM staging system for colon cancer [9]. Therefore, to achieve a more personalized prognosis for colon cancer patients, incorporating more prognostic factors in addition to current clinical prognostic factors would be a considerable choice.

Incorporating molecular factors, such as gene expression data, would be a considerable option for improving the performance of colon cancer prognosis. However, in gene expression-based analyses of heterogeneous diseases, a single gene often provides weak information [10]. However, the gene set obtained directly by analyzing a large number of genes is not stable and will change with changes in the training samples [11]. Several studies about the prognosis of colon cancer tried to select hypoxia-related genes or tumor microenvironment-related genes through literature reviews, but further screenings of these selected genes were still required in subsequent prognostic analyses [12, 13]. Therefore, the introduction of representative functional units, such as gene sets or pathways, may yield a more stable performance and may simultaneously provide certain biological annotations to improve the interpretability of the results [14–17]. In addition, converting the gene expression profile into personalized pathway activities showed a better prediction performance than using the origin gene expression profile in previous studies [18, 19].

In recent years, machine learning methods have been widely used for cancer prognostic analysis. When performing prognostic analyses through machine learning methods, the introduction of prior knowledge, such as pathway information, can further improve the performance of the model [20]. In most associated studies, molecular prognostic features were obtained by considering only the molecular features; therefore, new molecular features obtained through analysis may not be effectively combined with clinical features [21].

In this study, we conducted a knowledge-based clinical-molecular integrated analysis through a machine learning method, identified new pathway-based molecular prognostic factors to supplement the clinical TNM staging system for colon cancer overall survival prognosis prediction, and verified the improved performance of the clinical-molecular integrated prognostic models compared to the clinical prognostic model.

## 2. Materials and Methods

### 2.1. Data Acquisition and Processing

Gene mRNA expression data from primary tumors and related clinical data of 452 patients in The Cancer Genome Atlas Colon Adenocarcinoma (TCGA-COAD) project were obtained from cBioPortal as the discovery set, and gene expression data from normal adjacent tissues of 41 patients in the TCGA-COAD were obtained from the UCSC Xena as the reference set [22]. The mRNA sequence data of the discovery set and reference set used in this study were generated with the Illumina HiSeq 2000 platform and processed by the RNAseqV2 pipeline, which uses RNA-Seq by expectation maximization upper quartile (RSEM-UQ) for quantification. To validate the prognostic performance of the identified pathway-based factors, one independent dataset that offered identical clinical data and gene mRNA expression from primary tumors generated with a similar pipeline of 106 colon cancer patients was obtained from the Clinical Proteomic Tumor Analysis Consortium (CPTAC) from the LinkedOmics as the validation set [23]. The mRNA sequence data of the validation set used in this study were generated with the Illumina HiSeq 4000 platform and processed by the RNAseqV2 pipeline with RSEM-UQ for quantification. Both datasets can be used for an integrated analysis of clinical data and omics data.

Patients with primary tumors with both clinical data and gene expression data in the discovery set and validation set were included in this study. All data were cleaned and checked after data acquisition. The clinical data included T, N, and M stages and overall survival information. Other clinical prognostic factors, such as age and location, were not included because this study is focused on supplementing the clinical TNM staging system. The T stage was categorized into T1, T2, T3, and T4 stages (1 = T1, 2 = T2, 3 = T3, and 4 = T4 in subsequent analyses); the N stage was categorized into N0, N1, and N2 stages (0 = N0, 1 = N1, and 2 = N2 in subsequent analyses); and the M stage was categorized into M0 and M1 stages (0 = M0 and 1 = M1 in subsequent analyses). All gene expression data values were further log-transformed (Log2 (value + 1)) for subsequent analysis.

The following exclusion criteria were applied to the samples: containing Tis, N1c, or MX; lack of clear T, N, and M stages; and invalid survival information. In gene expression data, genes that could not be targeted with accurate HUGO Gene Nomenclature Committee (HGNC) symbols in the discovery set, validation set, and reference set were removed. Besides, genes with missing expression values or zero values were removed as well.

### 2.2. Study Design

**Figure 1:**
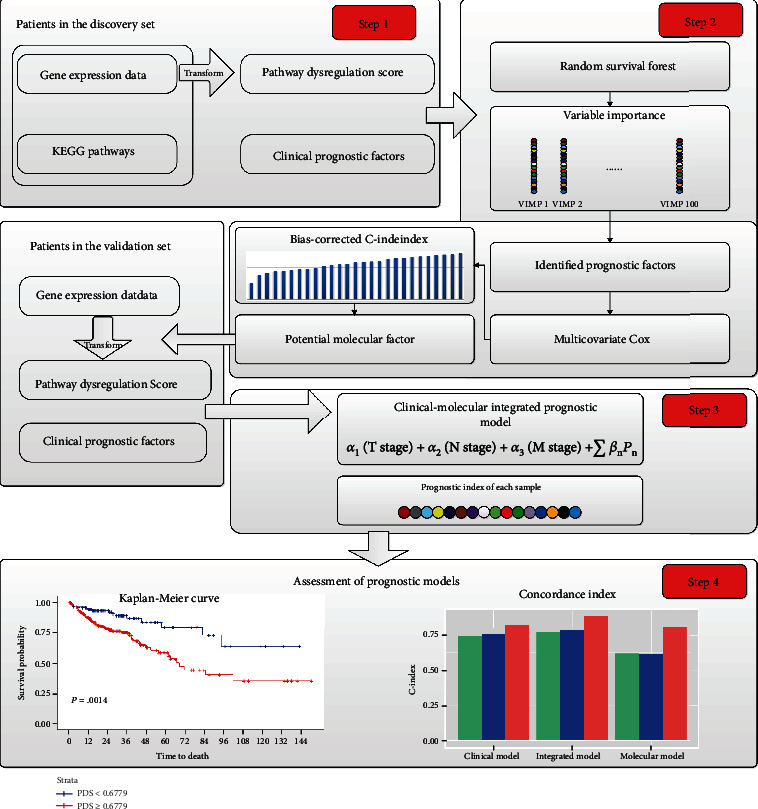
The overall pipeline of the study. Step 1: calculation of PDS; step 2: identification of molecular prognostic factors; step 3: construction of clinical-molecular integrative prognostic model; step 4: assessment of the integrative prognostic model.

First, we converted the gene expression data into pathway dysregulation scores (PDSs) based on prior knowledge from the Kyoto Encyclopedia of Genes and Genomes (KEGG) human pathway database. Then, we conducted a clinical-molecular integrated analysis by combining machine learning methods and survival analysis to identify the best molecular prognostic factors among the converted pathway-based factors. Finally, a clinical-molecular integrated prognostic model was constructed using clinical factors and the identified molecular factors for overall survival prediction and compared with the corresponding clinical prognostic model. The overall pipeline of this study is shown in Figure 1.

### 2.3. PDS Calculation

Among the pathway-based approaches, two methods, PARADIGM and Pathifier, are widely used to estimate the pathway dysregulation information in a particular sample [24, 25]. However, PARADIGM requires pathway mechanisms and is inappropriate for complex or incomplete pathways. Pathifier requires only the expression data of genes involved in each pathway and is more suitable for this study. In addition, previous studies confirmed that the PDS calculated by this method can effectively characterize pathway abnormalities [17, 19, 24, 26]. The PDS quantifies the biological difference of a specific pathway between a diseased sample and normal samples with a numeric value range from 0 to 1, and it is transformed from the gene expression data by the R package Pathifier [24]. The PDS in each sample indicates the distance of deviation between the projection of a specific pathway and the projection of normal samples on the principle component curve. The pathway information was obtained from KEGG with the R package KEGGREST (version 1.26.1).

In this study, the PDSs of 327 human pathways obtained from KEGG were calculated based on this method.

### 2.4. Identification of Molecular Prognostic Factors

In this study, the random survival forest was used to screen prognostic factors that could supplement clinical prognosis, and then the multicovariate Cox model was used to identify prognostic factors that could be the best supplementary factors for clinical prognostic factors.

#### 2.4.1. Identification with Random Survival Forest

The random survival forest is an ensemble tree-based method used to analyze right-censored survival data [27]. The nonparametric random survival forest model can assess the nonlinear effects of variables and explore the complex interactions between variables. In addition, variables in the random survival forest model that do not have prognostic ability can be filtered by variable importance. The variable selection procedure through the random survival forest in this study consists of the following three steps:

- Construct a random survival forest model with candidate variables. The numbers of trees that offer the lowest error rate were chosen
- In the constructed random survival forest model, variables with importance greater than 0 are selected and recorded
- Considering the existence of random processes, steps A and B would be repeated 100 times to generate a matrix of variables with a variable importance value greater than 0. The prognostic factors that were recorded as important prognostic factors multiple times were regarded as important prognostic factors

In this study, identification of molecular prognostic factors through the random survival forest was implemented with the following procedures. First, we performed a rough screening on all molecular factors. The clinical prognostic factors and all molecular factors were used as variables in the random survival forest. Variables that showed positive prognostic power more than 90 times according to the variable selection procedure were identified as the potential important prognostic factors. Then, we tried to identify robust molecular prognostic factors that could supplement the clinical prognostic factors. The potential important prognostic factors identified by the rough screening were screened again. Here, the identified potential important molecular factors and the clinical prognostic factors were used as variables of the random survival forest. The variable selection procedure was repeated 10 times to ensure the robustness. In each repetition, variables that showed positive prognostic power over 95 times were recorded as important prognostic factors. Finally, molecular factors that were recorded as important prognostic factors in all 10 repetitions were regarded as the final important prognostic factors identified by the random survival forest.

#### 2.4.2. Identification with Multicovariate Cox Model

Multicovariate Cox models were constructed to identify the best molecular factors for clinical prognostic supplementation. These models were constructed with clinical prognostic factors and different combinations of molecular prognostic factors identified in Section 2.4.1. The models in which molecular factors showed no statistical significance of prognostic importance (with a *P* value of the covariate larger than 0.05) were excluded. The discrimination performance of the remaining models was measured by the bias-corrected concordance index (C-index). Molecular prognostic factors in the model with the best discrimination performance were regarded as the best molecular prognostic factors. If multiple models showed similar discrimination performance, the molecular factors that used the least number of genes were regarded as the best molecular prognostic factors.

### 2.5. Construction of the Clinical-Molecular Integrated Prognostic Model

The clinical prognostic factor T, N, and M stages and the identified best molecular prognostic factors were used to construct the clinical-molecular integrated prognostic model. Therefore, a multicovariate Cox model was built, with the formula as follows:

(1) $$\begin{array}{c} h\left(t\right)=h\left(0\right)\exp\left(\alpha_{1}\left(\text{T stage}\right)+\alpha_{2}\left(\text{N stage}\right)+\alpha_{3}\left(\text{M stage}\right)+\sum \beta_{n}M_{n}\right), \end{array}$$

where *h*(*t*) is the risk of death at time *t*, *h*(0) is the baseline risk, *α* is the regression coefficient of clinical prognostic factors, *β* is the regression coefficient of molecular prognostic factors, and *M* is the identified molecular prognostic factor. In addition, identical clinical prognostic factors and molecular prognostic factors were used to construct the corresponding clinical prognostic model and molecular prognostic model. Comparisons of these models were performed to evaluate the improvement of prognostic performance between the clinical-molecular integrated prognostic model and the clinical prognostic model. Finally, a nomogram was constructed based on the clinical-molecular integrated prognostic model to predict the 3-year colon cancer overall survival.

### 2.6. Assessment of the Clinical-Molecular Integrated Prognostic Model

First, according to the distribution of the PDS of the corresponding molecular factors identified, patients in the discovery set were divided into different groups. The grouping was based on the highest degree of differentiation of survival curves. These findings could provide a direct observation of the relevance of the identified molecular prognostic factors and survival.

Second, based on the clinical prognostic factors and identified molecular prognostic factors, one clinical prognostic model, one molecular prognostic model, and one clinical-molecular integrated prognostic model were constructed on the discovery set. Internal validation through bootstrapping with 200 iterations was used to assess the discrimination performance of these models on the discovery set. External validation through stratified bootstrapping with 200 iterations was used to assess the discrimination performance of these models on the validation set. As the mean survival time of patients with metastasis was shorter than that of patients without metastasis, the performance of the prognostic model might have been affected. Therefore, models for nonmetastatic patients were built with the same prognostic factors and compared with the same assessment.

Finally, to compare the prognostic performance of directly using gene expression data and using converted PDS in this study, genes involved in the pathways were combined with clinical prognostic factors in the clinical-molecular integrated prognostic model. Comparisons between the gene-based integrated prognostic model and pathway-based integrated prognostic model were conducted.

The constructed integrated prognostic model had a potential problem of overfitting as it contains multiple covariates. The bias-corrected Harrell's C-index which overcomes the problem of overfitting was chosen to evaluate the overall discriminative performance of the models in internal validation [28]. The origin Harrell's C-index was used in external validation of the overall discriminative performance. Uno's C-index, which is free of censoring, was chosen to evaluate the discriminative performance of the models at the 3-year time point [29]. A two-sided Wilcoxon signed-rank test was used to compare the 200 C-indexes generated from the 200 iterations of the bootstrapping procedure to quantify the discriminative difference of the C-index between different models.

### 2.7. Statistical Analysis

All statistical analyses were performed using R statistical software (version 3.5.3). Construction of Cox models and the nomogram and internal validation of Harrell's C-index and calibration plot were performed with the rms R package. External validation of Harrell's C-index was performed with the Hmisc R package. Uno's C-index was calculated with the survC1 R package. The Wilcoxon signed-rank test was performed with the stats R package. The random survival forest was performed with the randomForestSRC R package.

## 3. Results

### 3.1. Results of Data Processing

After data acquisition and processing, this study included 374 cases in the TCGA-COAD data as the discovery set, 98 colon cancer cases in the CPTAC as the validation set, and 41 colon cancer normal adjacent tissue data from the TCGA as the reference set. Both the discovery set and the validation set included the T, N, and M stages with identical categories and overall survival information including overall survival time and overall survival status. The detailed information of the final dataset used for analysis is shown in Table 1.

### 3.2. Identification of Molecular Prognostic Factors

Through the random survival forest, a total of 14 pathways were screened as potential molecular prognostic factors as shown in Table 2. After further screening through the multicovariate Cox model, 27 combinations of different pathways were found to have significant prognostic effects in the integrated models. Based on the bias-corrected C-indexes of these 27 different clinical-molecular integrated models shown in Table 3, and the numbers of genes used in the analysis of each pathway shown in Table 2, we concluded that the PDS of the pathway has00532 should be the best molecular prognostic factor to supplement clinical prognosis among these 14 pathways. In this study, 16 genes were included in the analysis: XYLT1, XYLT2, B4GALT7, B3GALT6, B3GAT3, CSGALNACT1, CSGALNACT2, CHSY1, CHPF, CHPF2, DSE, CHST11, CHST12, CHST3, CHST15, and CHST14. The other 4 genes, CHSY3, CHST13, CHST7, and UST, were removed during data processing as these four genes were not matched in the validation set. These 16 genes were used for gene-based model construction in subsequent analyses.

**Figure 2:**
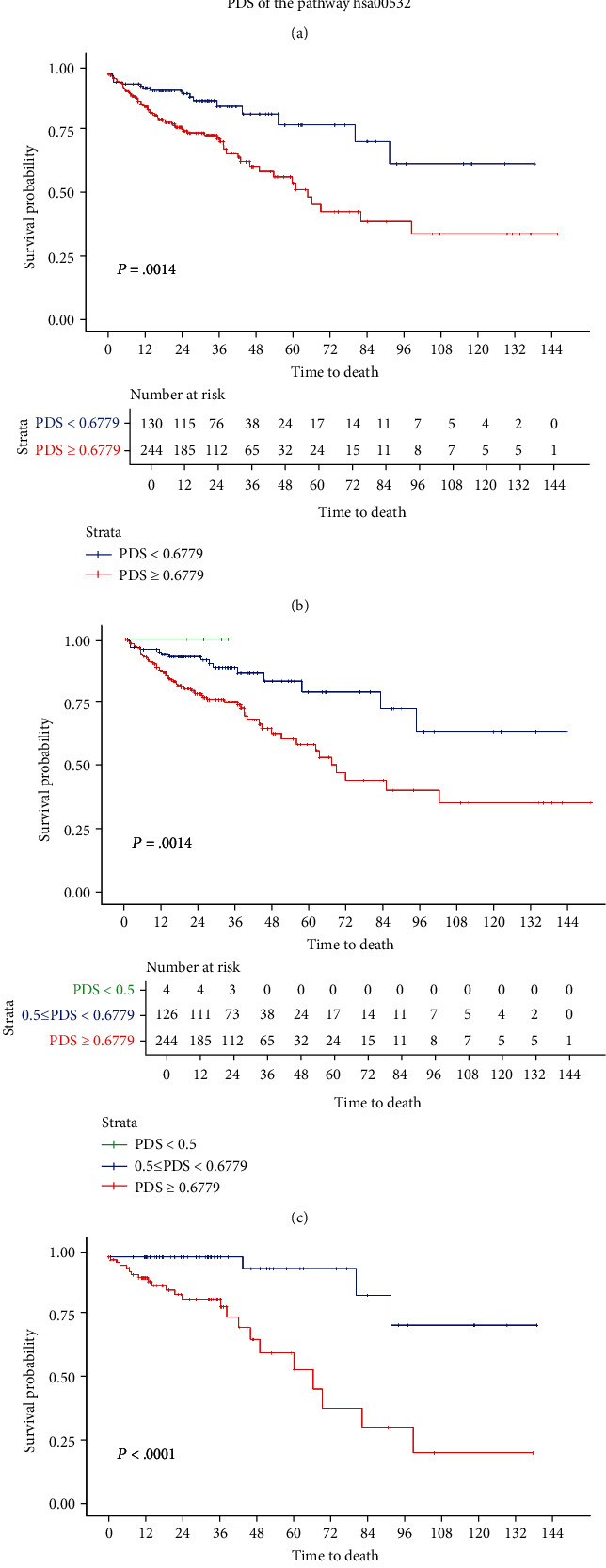
Observation of the PDS of the pathway hsa00532 in the discovery set. (a) Density distribution of the PDS of the pathway hsa00532 in the discovery set. (b) KM curve plotted based on two groups of patients in the discovery set divided by the PDS with a threshold of 0.6779. (c) KM curve plotted based on three groups of patients in the discovery set divided by the PDS with thresholds of 0.5 and 0.6779. (d) KM curve plotted based on two groups of stage II patients in the discovery set divided by the PDS with a threshold of 0.6779.

Observation of the distribution of the PDS of the pathway hsa00532 in the discovery set suggested that it approximately obeyed a normal distribution as shown in Figure 2(a). Therefore, patients in the discovery set were divided into a high-PDS group and a low-PDS group. Based on the difference in Kaplan-Meier (KM) curves between different patient groups, a threshold of 0.6779 was considered to most clearly separate these two groups, with the corresponding KM curves shown in Figure 2(b). In addition, several peaks at approximately less than 0.5 of the density distribution led us to separate the patients into three groups according to thresholds of 0.5 and 0.6779, with the corresponding KM curves shown in Figure 2(c). The high-PDS and low-PDS groups divided by 0.6779 showed significant survival differences in stage II colon cancer patients, as shown in Figure 2(d).

### 3.3. Constructed Knowledge-Based Clinical-Molecular Integrated Prognostic Model

**Figure 3:**
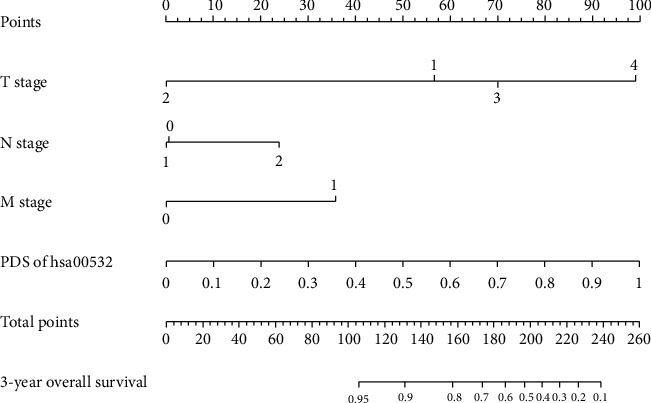
Nomogram for predicting the 3-year overall survival of colon cancer patients. To use the nomogram, first, the position of each variable of an individual patient on the corresponding axis should be found. Next, a line to the point axis for the number of points should be drawn upwards to determine the number of points of each variable. Then, the points from all the variables should be added. Finally, a line from the total point axis should be drawn downward to determine the likelihood of 3-year survival probabilities at the lower line of the nomogram.

With the identified knowledge-based prognostic factor, the PDS of the pathway hsa00532, and clinical prognostic factor T, N, and M stages, our knowledge-based clinical-molecular integrated prognostic model was built. To assess the improvement of our model compared with the clinical prognostic model, the corresponding clinical prognostic model based on T, N and M stages and the molecular prognostic model based on the PDS of pathway has00532 were constructed. The multicovariate Cox model was used to determine the regression coefficients of the models, with the coefficients of the knowledge-based clinical-molecular integrated prognostic model summarized in Table 4 and regression coefficients of the other models summarized in Table S1, Table S2, and Table S3. A corresponding nomogram that predicts the 3-year overall survival was constructed and is shown in Figure 3.

### 3.4. Assessment of the Prognostic Models for all Colon Cancer Patients

**Figure 4:**
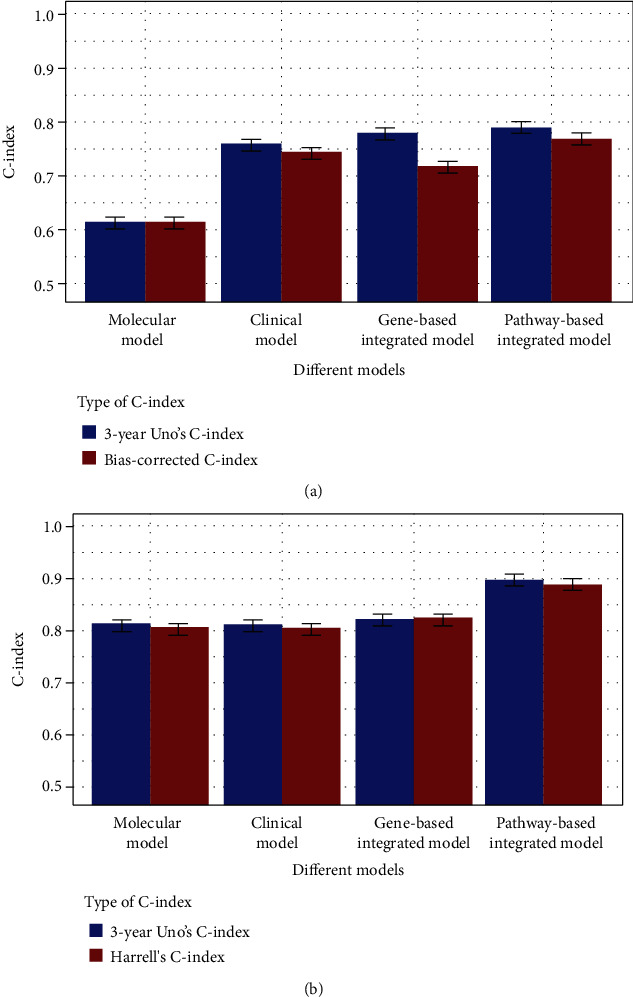
C-index of our pathway-based integrated model and other models for patients in the discovery set (a) and validation set (b).

The discriminative performance of different models was measured with both Harrell's C-index for overall performance and 3-year Uno's C-index for performance at specific time points and is shown in Figure 4. In the internal validation, our model outperformed in terms of overall prognostic performance compared to the clinical prognostic model (0.773 vs 0.746, *P* < .001) and the molecular prognostic model (0.773 vs 0.619, *P* < .001) as shown in Figure 4(a). In the external validation, our model again outperformed in terms of overall prognostic performance compared to the clinical prognostic model (0.893 vs 0.808, *P* < .001) and the molecular prognostic model (0.893 vs 0.810, *P* < .001) as shown in Figure 4(b).

**Figure 5:**
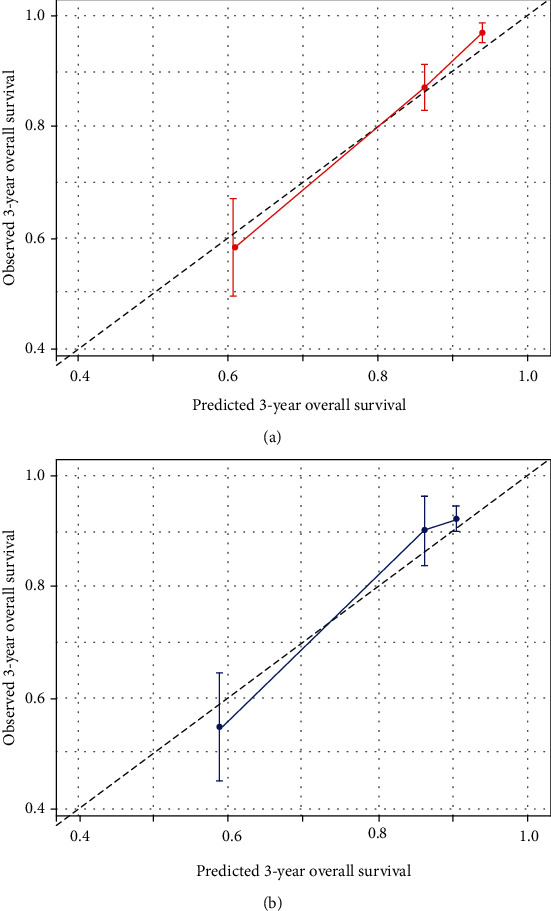
Calibration plot of our pathway-based integrated model (a) and clinical model (b) at the 3-year time point.

The prognostic performance of our model at the 3-year time point was assessed by Uno's C-index and calibration plot. The 3-year Uno's C-index in the discovery set suggested that our model has the best discriminative performance compared to the clinical model (0.793 vs. 0.762, *P* < .001) and the molecular model (0.793 vs. 0.619, *P* < .001) as shown in Figure 4(a), whereas in the validation set, the comparison results were 0.899 vs. 0.816 (*P* < .001) compared to the clinical model and 0.899 vs. 0.816 (*P* < .001) compared to the molecular model as shown in Figure 4(b). The calibration plot of these models also showed that our model has a superior calibration performance compared with the clinical model at a 3-year time point as shown in Figure 5.

### 3.5. Assessment of the Prognostic Models for Nonmetastatic Colon Cancer Patients

**Figure 6:**
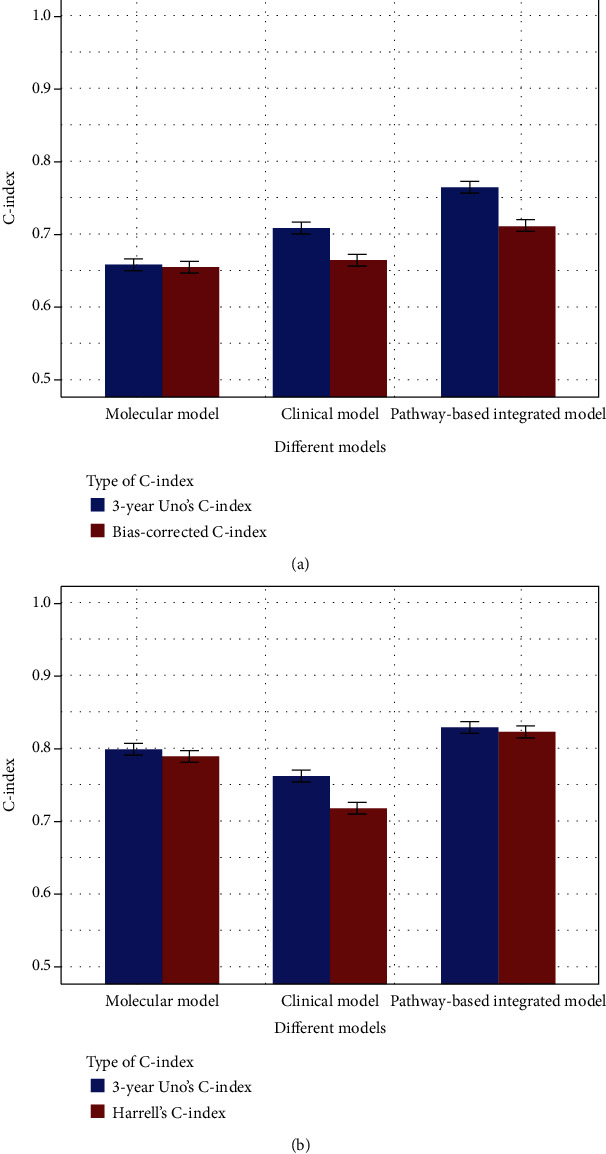
C-index of our pathway-based integrated model and other models for nonmetastatic patients in the discovery set (a) and the validation set (b).

Because the mean survival time of metastatic patients is shorter than that of nonmetastatic patients, the prognostic performance of the prognostic models may be affected. Therefore, the clinical prognostic model, molecular prognostic model, and clinical-molecular integrated prognostic model were constructed with the same prognostic factors used for nonmetastatic patients. The same assessments were performed on these models for nonmetastatic patients, with nonmetastatic patients in the discovery set and the validation set. In the internal validation, the integrated model outperformed in terms of overall prognostic performance compared to the clinical prognostic model (0.712 vs. 0.665, *P* < .001 for bias-corrected Harrell's C-index, 0.763 vs. 0.709, *P* < .001 for the 3-year Uno's C-index) and the molecular prognostic model (0.712 vs. 0.655, *P* < .001 for bias-corrected Harrell's C-index, 0.763 vs. 0.659, *P* < .001 for the 3-year Uno's C-index) as shown in Figure 6(a). In the external validation, the integrated model again outperformed in terms of overall prognostic performance compared to the clinical prognostic model (0.824 vs. 0.720, *P* < .001 for Harrell's C-index, 0.829 vs. 0.743, *P* < .001 for the 3-year Uno's C-index) and the molecular prognostic model (0.824 vs. 0.791, *P* < .001 for Harrell's C-index, 0.829 vs. 0.799, *P* < .001 for the 3-year Uno's C-index) as shown in Figure 6(b).

### 3.6. Pathway-Based Model Is Superior to the Gene-Based Model

Previous studies have claimed that the introduction of representative functional units should improve gene expression-based studies [10, 14–16, 30]. Therefore, genes involved in the pathway hsa00532 were used to construct a gene-based clinical-molecular integrated prognostic model. The regression coefficients of the gene-based model suggested that only two genes (CSGALNACT1 and DSE) were prognostically related when combined with clinical factors, and they are summarized in Table S3. Compared with our knowledge-based integrated model, the C-indexes of the gene-based integrated model in the discovery set were lower, with 0.721 vs. 0.773 (*P* < .001) for the bias-corrected C-index, 0.783 vs. 0.793 (*P* < .001) for the 3-year Uno's C-index in the discovery set, 0.825 vs. 0.893 (*P* < .001) for Harrell's C-index, and 0.826 vs. 0.899 (*P* < .001) for the 3-year Uno's C-index in the validation set as shown in Figure 4. These results suggest that the pathway-based integrated model is superior to the gene-based integrated model in discriminative performance because the gene-based integrated model might include too many redundant prognostic factors.

## 4. Discussion

### 4.1. Principal Results

Through knowledge-based clinical-molecular integrated analysis by the random survival forest and multicovariate Cox model, this study successfully identified the PDS of the pathway hsa00532 as the best molecular prognostic factor for supplementing the prognostic performance of the T, N, and M stages in overall survival prediction. The results of internal validation and external validation suggested that the knowledge-based clinical-molecular integrated prognostic model had the best discriminative performance and improved calibration performance than the clinical prognostic model. The regression coefficients of the covariate in different models in Table 4, Table S1, and Table S2 indicated that tiny changes were observed for the clinical prognostic factors, while the molecular prognostic factor keeps as an independent prognostic factor in the final clinical-molecular integrated prognostic model. These further indicated that our final clinical-molecular integrated prognostic model does satisfy the aim of our study. In addition, the pathway-based models were superior to the gene-based model, which indicates that the incorporation of pathway information can make more use of the expression information of genes involved in a pathway rather than directly using the expression information of genes.

The observation of the KM curves based on patient groups divided by thresholds of 0.5 and 0.6779 suggested that patients with higher PDSs had worse survival. In addition, the KM curves of stage II patients divided into high-PDS and low-PDS groups could be distinctly distinguished, indicating that the PDS of the pathway hsa00532 might be a potential biomarker for separating high-risk stage II colon cancer patients.

The pathway hsa00532 is named glycosaminoglycan biosynthesis-chondroitin sulfate/dermatan sulfate on the KEGG website, and it is related to the biosynthesis of chondroitin sulfate and dermatan sulfate. Previous studies have indicated that the dermatan sulfate chain is different between colon cancer and normal colonic mucosa, and chondroitin sulfate is associated with tumor metastasis [31–34]. However, the PDS of the pathway hsa00532 showed no relevance to the metastasis status, with a Pearson correlation coefficient of 0.04 for the discovery dataset. In addition, the pathway hsa00532 showed considerable supplementary power in the models for both all-stage and nonmetastatic colon cancer patients, while the metastasis status and the PDS of the pathway hsa00532 were regarded as independent significant prognostic factors in the constructed model. One possible explanation for this finding is that although the PDS in this study is generated from gene expression data, a series of regulatory and expression biology processes from the transcriptome is still needed to generate the actual pathway products. Two genes, CSGALNACT1 and DSE, involved in the pathway hsa00532 might be potential markers for colon cancer prognosis because only these two genes showed a potential prognostic effect in the gene-based integrated model based on the regression coefficients of the gene-based model summarized in Table S3. Further validation is required to validate the prognostic effect of these two genes as currently published papers have not mentioned them in conjunction with colon cancer prognosis.

Recent studies on colon cancer prognosis mainly focused on finding better molecular prognostic features, while our study was aimed at supplementing the current clinical staging system with molecular features [12, 13, 35]. Compared to a recent colon cancer prognosis study which incorporated both clinical prognostic features and gene expression profiles, our study integrated the clinical prognostic features and gene expression profile in a conditional way rather than joining the two types of features independently [12]. The conditional modelling strategy is more suitable for our study as this study was aimed at supplementing the prognosis performance of the current TNM staging system.

### 4.2. Limitations

There are still limitations in this study. The clinical prognostic factors in this study involved only the T, N, and M stages, while in the actual clinical treatment of colon cancer, there are many other factors that need to be considered, such as the patient's physical condition and the chemotherapy or radiotherapy regimen. In addition, due to the short follow-up time of the validation set, it was not possible to further validate the performance of our model on long-term prognosis. The conditional modelling strategy for clinical-molecular integration could satisfy the demand of the current study, although it could not fully utilize the correlation structure between clinical and molecular factors [36]. Further studies about how to make better use of molecular features should be considered. The current study used only gene expression data from the transcriptome, and the addition of other types of omics data, such as genome or epigenome data, may further improve the accuracy of molecular features and better supplement the clinical prognosis. However, with the current technology, how to balance the improvement in discriminative performance and the cost of sequencing remains to be considered.

Through cooperation with local hospitals, we can collect more real-world follow-up patients and sequence their tumor samples to generate more molecular data. Therefore, further validation of our model could be conducted. The involvement of more clinical prognostic factors in clinical-molecular analysis could make a more detailed and specific supplement to clinical prognosis. New integrative models based on a conditional strategy or even a joint modelling strategy would be required to deal with new data. In addition, considering that the PDS of the pathway hsa00532 can effectively distinguish the risk of stage II patients, further research and validation should be performed with more data. After further validation with real data, further research related to the PDS of the pathway has00532, such as immunohistochemistry and other methods appropriate for clinical use, should be conducted, with corresponding web-based tools being developed.

## 5. Conclusions

In conclusion, this study identified that the PDS of the pathway hsa00532 can be used as a supplementary prognostic factor for the three clinical prognostic factor T, N, and M stages. The clinical-molecular integrated prognostic model constructed with these three clinical prognostic factors and the identified molecular prognostic factor is superior to the clinical prognostic model, molecular prognostic model, or gene-based integrated prognostic model in prognostic performance. A corresponding nomogram including the three clinical prognostic factors and the identified molecular prognostic factor was constructed for possible clinical use. In addition, the PDS of the pathway hsa00532 showed a significant ability to distinguish high risk stage II colon cancer patients and is a potential prognostic marker. The PDS calculation of the pathway hsa00532 involves only 16 genes; therefore, it has good prospects for clinical use after further validation with real data.

## Figures and Tables

**Table 1:** Detailed information of the data used for analysis.

Characteristic	Discovery set	Validation set	Reference set
	TCGA-COAD	CPTAC	Normal samples
Patients, *n*	374	98	41
Survival status, *n* (%)			
Alive	293 (78.3)	90 (91.8)	29 (70.7)
Dead	81 (21.7)	8 (8.2)	12 (29.3)
Age^a^ in years, mean (SD, range)	66.75 (12.73, 31-90)	65.43 (11.56, 35-93)	70.34 (13.23, 40-90)
Gender, *n* (%)			
Male	199 (53.2)	41 (41.8)	20 (48.8)
Female	175 (46.8)	57 (58.2)	21 (51.2)
Overall survival time in months, mean (median, range)	30.24 (24.27, 0.47-150.07)	27.96 (30, 1-44)	27.66 (24.37, 0-101.40)
T stage, *n* (%)			Not available
T1	9 (2.4)	0 (0)	
T2	65 (17.4)	12 (12.2)	
T3	258 (69.0)	73 (74.5)	
T4	42 (11.2)	13 (13.3)	
N stage, *n* (%)			Not available
N0	226 (60.4)	52 (53.1)	
N1	84 (22.5)	31 (31.6)	
N2	64 (17.1)	15 (15.3)	
M stage, n (%)			Not available
M0	315 (84.2)	91 (92.9)	
M1	59 (15.8)	7 (7.1)	
Number of genes, *n*	10877	10877	10877

^a^The characteristic “Age” refers to the age at initial diagnosis in the discovery set and reference set but refers to the age at procurement in the validation set. SD: standard deviation.

**Table 2:** Description of the pathways identified by the random survival forest.

KEGG pathway ID	Pathway name	Number of genes involved in the pathway in this study
hsa00450	Selenocompound metabolism—Homo sapiens (human)	13
hsa00532	Glycosaminoglycan biosynthesis—chondroitin sulfate/dermatan sulfate—Homo sapiens (human)	16
hsa02010	ABC transporters—Homo sapiens (human)	24
hsa04380	Osteoclast differentiation—Homo sapiens (human)	105
hsa04614	Renin-angiotensin system—Homo sapiens (human)	14
hsa04750	Inflammatory mediator regulation of TRP channels—Homo sapiens (human)	65
hsa04911	Insulin secretion—Homo sapiens (human)	41
hsa04971	Gastric acid secretion—Homo sapiens (human)	40
hsa04975	Fat digestion and absorption—Homo sapiens (human)	13
hsa05032	Morphine addiction—Homo sapiens (human)	38
hsa05133	Pertussis—Homo sapiens (human)	56
hsa05152	Tuberculosis—Homo sapiens (human)	128
hsa05167	Kaposi sarcoma-associated herpesvirus infection—Homo sapiens (human)	148
hsa05321	Inflammatory bowel disease (IBD)—Homo sapiens (human)	34

**Table 3:** Bias-corrected C-indexes of 27 different clinical-molecular integrated models.

Covariates used in the model	Bias-corrected Harrell's C-index (±95% CI)
T, N, M, hsa00532, hsa04911, hsa05133, hsa05152	0.775 ± 0.0038
T, N, M, hsa00532	0.773 ± 0.0038
T, N, M, hsa00532, hsa04911, hsa05133	0.773 ± 0.0038
T, N, M, hsa02010, hsa05152, hsa05321	0.773 ± 0.0038
T, N, M, hsa02010, hsa05167, hsa05321	0.772 ± 0.0037
T, N, M, hsa00532, hsa04380, hsa04911, hsa05133	0.772 ± 0.0039
T, N, M, hsa00532, hsa05133, hsa05152	0.772 ± 0.0040
T, N, M, hsa00532, hsa04380, hsa04971, hsa05133	0.771 ± 0.0039
T, N, M, hsa00532, hsa05133, hsa05167	0.771 ± 0.0040
T, N, M, hsa00532, hsa04975, hsa05133	0.770 ± 0.0041
T, N, M, hsa02010, hsa04911, hsa05133, hsa05152	0.768 ± 0.0040
T, N, M, hsa02010, hsa05133, hsa05152	0.768 ± 0.0040
T, N, M, hsa00532, hsa04971, hsa05133	0.767 ± 0.0038
T, N, M, hsa00532, hsa04380, hsa05133	0.766 ± 0.0041
T, N, M, hsa02010, hsa04911, hsa05133	0.764 ± 0.0038
T, N, M, hsa02010, hsa05133, hsa05167	0.764 ± 0.0040
T, N, M, hsa00532, hsa04750, hsa05133	0.764 ± 0.0041
T, N, M, hsa02010, hsa04911	0.763 ± 0.0037
T, N, M, hsa02010, hsa04380, hsa04911, hsa05133	0.763 ± 0.0040
T, N, M, hsa05133, hsa05152	0.761 ± 0.0042
T, N, M, hsa02010, hsa04750, hsa05133	0.759 ± 0.0040
T, N, M, hsa00450, hsa04911	0.758 ± 0.0036
T, N, M, hsa04380, hsa05133	0.758 ± 0.0043
T, N, M, hsa04911, hsa05133	0.756 ± 0.0039
T, N, M, hsa00450, hsa04911, hsa05133, hsa05152	0.756 ± 0.0040
T, N, M, hsa04380, hsa04911, hsa05133	0.754 ± 0.0041
T, N, M, hsa05133, hsa05167	0.754 ± 0.0042
T, N, M	0.746 ± 0.0040

CI: confidence interval.

**Table 4:** Regression coefficients of the knowledge-based clinical-molecular integrated prognostic model.

Covariate	Coefficient ± SE	HR	95% CI	*P* value
T stage				
T2	−1.62 ± 1.42	0.20	0.012-3.18	.25
T3	0.38 ± 1.02	1.47	0.20-10.79	.71
T4	1.22 ± 1.05	3.38	0.43-26.37	.25
N stage				
N1	−0.01 ± 0.31	0.99	0.54-1.80	.96
N2	0.67 ± 0.30	1.95	1.07-3.54	.03
M stage				
M1	1.02 ± 0.28	2.78	1.60-4.82	<.001
hsa00532^∗^	2.86 ± 1.42	17.53	1.08-283.24	.04

SE: standard error; HR: hazard ratio; CI: confidence interval. ^∗^Covariate hsa00532 used in the model is the PDS of pathway has00532.

## Data Availability

The dataset analyzed during the current study is available in the cBioPortal, http://www.cbioportal.org; generated by the National Cancer Institute CPTAC and available in the LinkedOmics, http://linkedomics.org/cptac-colon/ and available in the UCSC Xena, http://xena.ucsc.edu/.

## Supplementary Materials

Supplementary 1 Table S1: regression coefficients of the clinical prognostic model.

Supplementary 2 Table S2: regression coefficients of the molecular prognostic model.

Supplementary 3 Table S3: regression coefficients of the gene-based clinical-molecular integrated prognostic model.
