# Supplementary material for: Improving the Prognosis of Colon Cancer through Knowledge-Based Clinical-Molecular Integrated Analysis
Source: Biomed Res Int. 2021 Apr 7;2021:9987819. doi: 10.1155/2021/9987819 (PMC8051523; doi:10.1155/2021/9987819)
Supplement: Supplementary 1 — Table S1: regression coefficients of the clinical prognostic model. [file 9987819.f1.docx]

Supplementary Table S1

**Table S1 Regression coefficients of the clinical prognostic model.**

| Covariate | Coefficient ± SE | HR | 95% CI | P value |
| --- | --- | --- | --- | --- |
| T Stage  T2  T3  T4 | -1.65 ± 1.42  0.39 ± 1.02  1.19 ± 1.05 | 0.19  1.47  3.29 | 0.012-3.10  0.20-10.83  0.42-25.75 | 0.25  0.70  0.26 |
| N Stage  N1  N2 | 0.06 ± 0.31  0.75 ± 0.30 | 1.06  2.11 | 0.58-1.94  1.17-3.82 | 0.85  0.014 |
| M Stage  M1 | 1.01 ± 0.28 | 2.75 | 1.59-4.77 | 0.00030 |

SE: standard error; HR: hazard ratio; CI: confidence interval
