# Supplementary material for: Improving the Prognosis of Colon Cancer through Knowledge-Based Clinical-Molecular Integrated Analysis
Source: Biomed Res Int. 2021 Apr 7;2021:9987819. doi: 10.1155/2021/9987819 (PMC8051523; doi:10.1155/2021/9987819)
Supplement: Supplementary 2 — Table S2: regression coefficients of the molecular prognostic model. [file 9987819.f2.docx]

Supplementary Table S2

**Table S2 Regression coefficients of the molecular prognostic model.**

| Covariate | Coefficient ± SE | HR | 95% CI | P value |
| --- | --- | --- | --- | --- |
| Hsa00532^*^ | 3.45 ± 1.33 | 31.42 | 2.30-428.90 | 0.0097 |

SE: standard error; HR: hazard ratio; CI: confidence interval

*The covariate Hsa00532 used in the model is the PDS of pathway has00532.
