# Supplementary material for: Improving the Prognosis of Colon Cancer through Knowledge-Based Clinical-Molecular Integrated Analysis
Source: Biomed Res Int. 2021 Apr 7;2021:9987819. doi: 10.1155/2021/9987819 (PMC8051523; doi:10.1155/2021/9987819)
Supplement: Supplementary 3 — Table S3: regression coefficients of the gene-based clinical-molecular integrated prognostic model. [file 9987819.f3.docx]

Supplementary Table S3

**Table S3 Regression coefficients of the gene-based clinical-molecular integrated prognostic model.**

| Covariate | Coefficient ± SE | HR | 95% CI | P value |
| --- | --- | --- | --- | --- |
| T Stage  T2  T3  T4 | -1.58 ± 1.43  0.38 ± 1.04  1.27 ± 1.09 | 0.21  1.47  3.57 | 0.013-3.39  0.19-11.27  0.43-29.95 | 0.27  0.71  0.24 |
| N Stage  N1  N2 | 0.03 ± 0.32  0.58 ± 0.34 | 1.03  1.79 | 0.55-1.93  0.92-3.48 | 0.92  0.087 |
| M Stage  M1 | 1.10 ± 0.31 | 3.01 | 1.64-5.53 | 0.00038 |
| XYLT1 | -0.14 ± 0.17 | 0.87 | 0.62-1.21 | 0.41 |
| XYLT2 | 0.23 ± 0.31 | 1.26 | 0.68-2.32 | 0.46 |
| B4GALT6 | -0.02 ± 0.29 | 0.98 | 0.55-1.74 | 0.94 |
| B3GALT7 | -0.29 ± 0.32 | 0.75 | 0.40-1.40 | 0.36 |
| B3GAT3 | 0.14 ± 0.34 | 1.15 | 0.59-2.24 | 0.69 |
| CSGALNACT1 | -0.50 ± 0.19 | 0.61 | 0.42-0.88 | 0.0079 |
| CSGALNACT2 | 0.11 ± 0.30 | 1.12 | 0.62-2.02 | 0.70 |
| CHSY1 | 0.18 ± 0.30 | 1.20 | 0.66-2.17 | 0.55 |
| CHPF | 0.14 ± 0.22 | 1.15 | 0.75-1.76 | 0.51 |
| CHPF2 | 0.31 ± 0.36 | 1.36 | 0.67-2.76 | 0.40 |
| DSE | 0.32 ± 0.19 | 1.37 | 0.95-1.99 | 0.094 |
| CHST11 | -0.10 ± 0.16 | 0.91 | 0.67-1.23 | 0.52 |
| CHST12 | -0.09 ± 0.29 | 0.91 | 0.52-1.60 | 0.74 |
| CHST3 | 0.04 ± 0.14 | 1.05 | 0.80-1.37 | 0.74 |
| CHST14 | 0.10 ± 0.26 | 1.10 | 0.67-1.83 | 0.71 |
| CHST15 | 0.09 ± 0.16 | 1.10 | 0.81-1.49 | 0.56 |

SE: standard error; HR: hazard ratio; CI: confidence interval
